# Defining severity of personality disorder using electronic health records: short report

**DOI:** 10.1192/bjo.2023.509

**Published:** 2023-08-01

**Authors:** Jonathan Monk-Cunliffe, Giouliana Kadra-Scalzo, Chloe Finamore, Oliver Dale, Mizanur Khondoker, Barbara Barrett, Hitesh Shetty, Richard D. Hayes, Paul Moran

**Affiliations:** Centre for Academic Mental Health, Department of Population Health Sciences, Bristol Medical School, University of Bristol, Bristol, UK; Department of Psychological Medicine, Institute of Psychiatry, Psychology and Neuroscience, King's College London, London, UK; Research Unit, The Cassel Hospital, West London NHS Trust, Richmond, UK; Norwich Medical School, University of East Anglia, Norwich, UK

**Keywords:** Economics, epidemiology, personality disorders, rating scales, physical health

## Abstract

Severity of personality disorder is an important determinant of future health. However, this key prognostic variable is not captured in routine clinical practice. Using a large clinical data-set, we explored the predictive validity of items from the Health of Nation Outcome Scales (HoNOS) as potential indicators of personality disorder severity. For 6912 patients with a personality disorder diagnosis, we examined associations between HoNOS items relating to core personality disorder symptoms (self-harm, difficulty in interpersonal relationships, performance of occupational and social roles, and agitation and aggression) and future health service use. Compared with those with no self-harm problem, the total healthcare cost was 2.74 times higher (95% CI 1.66–4.52; *P* < 0.001) for individuals with severe to very severe self-harm problems. Other HoNOS items did not demonstrate clear patterns of association with service costs. Self-harm may be a robust indicator of the severity of personality disorder, but further replication work is required.

People diagnosed with personality disorder have poorer health outcomes, impaired quality of life and reduced life expectancy compared with the general population and are more likely to be admitted to hospital for a variety of illnesses.^1–3^ The ICD-11 radically changed the classification of personality disorder to include the domain of severity (mild, moderate and severe). Other research has shown that severity of personality disorder carries important prognostic information.^4^ Yet, this key prognostic variable is not captured in routine clinical practice.^5^ The Health of the Nation Outcome Scales (HoNOS) is used widely in the UK as a measure of common clinical problems and social functioning and is used in the Payment by Results system.^6^ ICD-11 explicitly relates severity of personality disorder to risk of the individual to self and others. Furthermore, certain HoNOS items directly map on to some core personality disorder features – specifically the domains of self-harm, difficulty in interpersonal relationships, performance of occupational and social roles, and agitation and aggression. We aimed to investigate the association between personality disorder severity (as indexed by select HoNOS domains) and health service use, using routinely collected data.

## Method

### Setting

We conducted a retrospective cohort study using data from South London and Maudsley NHS Trust (SLaM), a large provider of secondary mental healthcare in South London, UK. We used the Clinical Record Interactive Search (CRIS) database to access anonymised electronic health record (EHR) information^7^ and identified patients with a personality disorder diagnosis receiving care between 1 January 2008 and 31 March 2018. We identified individuals with relevant ICD-10 codes in a structured diagnosis field and used natural language processing software to detect diagnoses in free-text fields, where information is often recorded in EHRs.^8^

### Exposures

HoNOS includes 12 items assessing common clinical problems and social functioning over the preceding 2 weeks.^6^ It is used widely in the UK and is available in EHRs; it was therefore adopted as an indicator of personality disorder severity (‘minor’ to ‘severe’). We included items 1 (harm to others), 2 (harm to self), 9 (interpersonal relationships) and 10 (performance of occupational and social roles) on an *a priori* basis, as they reflect core personality disorder symptoms. Observation began at the first HoNOS assessment in the study period.

### Outcomes

Total healthcare cost was used as a proxy measure of the intensity of health service use and to provide a summary value of the use of different types of service for analysis. We identified linked data from Hospital Episode Statistics (HES) on the number of general hospital in-patient admissions, bed days, out-patient appointments, and accident and emergency department (A&E) attendances in England and Wales. We attributed a cost to each in-patient bed day (£856.46), out-patient appointment (£125.01) and A&E attendance (160.32) using the UK's national schedule of reference costs.^9^ We added 0.5 bed days for individuals with ≥1 admission but 0 bed days to reflect individuals who may have been admitted and discharged on the same day.

### Statistics

We used Stata version 16 for all statistical analyses. We conducted a multivariable linear regression of the log-transformed total healthcare cost for each HoNOS item. Robust standard errors were used to minimise any potential impact of possible deviation of residual distribution from normality on statistical inference (Supplementary Appendix 1 available at https://doi.org/10.1192/bjo.2023.509). Coefficients were back-transformed to the original unit (£). We adjusted for years of follow-up, age, gender, deprivation, ethnicity, and comorbid psychosis or mood disorder (Supplementary Appendix 1). We checked the robustness of our findings by repeating the analyses, excluding individuals with zero cost and attributing different costs to each item. We also conducted a linear regression of cost with bootstrapped standard errors, as this uses the arithmetic mean, reflects the cost of treating all patients and is commonly used in health economic analyses.^10^

### Ethics statement

CRIS is approved as a data-set for secondary analysis (Oxfordshire Research Ethics Committee C, reference 08/H0606/71+5). All projects using the CRIS data resource are considered and approved by an oversight committee including patient representatives. The CRIS database contains anonymised EHR data and so no patient consent was required for this project.

## Results

We identified 6912 individuals meeting the inclusion criteria for the study (mean age 36.1 years, s.d. 13.4 years; 62% female; 67% White, 16.5% Black and 16.5% other ethnicity; 11% in a relationship; 38% with comorbid mood disorder and 23% with comorbid psychosis).

Table 1 displays results from the linear regression examining the relationship between each individual HoNOS item and the log-transformed total healthcare cost. The average healthcare cost for individuals with a severe to very severe self-harm problem was 2.74 times higher (95% CI 1.66–4.52; *P* < 0.001) than for individuals with no self-harm problem.

**Table 1:** Linear regression of the association between HoNOS items and log-transformed total healthcare cost

	Total healthcare cost			
	Unadjusted coefficient (95% CI)	*P*-value	Adjusted coefficient (95% CI)	*P*-value
Agitation and aggression				
Minor problem	1.16 (0.88–1.51)	0.290	1.16 (0.90–1.51)	0.255
Mild	0.85 (0.62–1.17)	0.321	1.07 (0.79–1.45)	0.674
Moderate	1.17 (0.78–1.76)	0.453	1.38 (0.93–2.06)	0.111
Severe to very severe	1.76 (0.99–3.13)	0.054	1.70 (0.99–2.92)	0.056
Self-harm				
Minor problem	1.13 (0.84–1.52)	0.422	1.41 (1.06–1.86)	0.018
Mild	1.15 (0.83–1.58)	0.400	1.84 (1.34–2.53)	<0.001
Moderate	1.04 (0.70–1.54)	0.850	1.80 (1.23–2.62)	0.002
Severe to very severe	1.88 (1.12–3.17)	0.018	2.74 (1.66–4.52)	<0.001
Relationship problems				
Minor problem	0.91 (0.66–1.26)	0.572	0.99 (0.72–1.35)	0.929
Mild	0.82 (0.61–1.12)	0.210	0.93 (0.69–1.25)	0.620
Moderate	0.65 (0.46–0.92)	0.015	0.90 (0.65–1.26)	0.548
Severe to very severe	0.82 (0.48–1.39)	0.456	1.04 (0.63–1.71)	0.878
Daily living problems				
Minor problem	1.63 (1.25–2.14)	<0.001	1.22 (0.94–1.58)	0.131
Mild	1.84 (1.35–2.50)	<0.001	1.33 (0.99–1.80)	0.058
Moderate	2.07 (1.28–3.35)	0.003	1.21 (0.76–1.93)	0.420
Severe to very severe	3.17 (1.31–7.69)	0.011	1.91 (0.77–4.72)	0.163

Reference category: no problem. Unadjusted models: aggression *n* = 6889, self-harm *n* = 6887, relationships *n* = 6864, daily living *n* = 6839. Adjusted models: aggression *n* = 6613, self-harm *n* = 6611, relationships *n* = 6590, daily living *n* = 6563.

Increasing severity of agitation and aggression was also associated with an increase in the coefficient for total healthcare cost. However, the confidence intervals around this estimate included no difference between the groups. In the adjusted models, other HoNOS items did not demonstrate a clear pattern of association with costs.

Sensitivity analyses revealed a similar pattern of results. The bootstrapped model showed similar results for self-harm and also found that increasing daily living problems were associated with increased costs (Supplementary Appendix 1).

## Discussion

Among individuals with a personality disorder diagnosis, the HoNOS self-harm domain appears to be independently associated with future secondary acute health service use (as indexed by cost). Our measure of cost was 2.74 times higher (95% CI 1.66–4.52; *P* < 0.001) for individuals with severe self-harm compared with individuals with no self-harm problem. This HoNOS domain has previously been linked to future healthcare costs, including in a small sample of Norwegian patients with borderline personality disorder.^11,12^ We have now demonstrated the utility of this HoNOS domain as an indicator of personality disorder severity in a much larger sample. The other HoNOS domains did not show clear associations with secondary health service use. Although we note for severe to very severe agitation and aggression, the cost was 1.70 times higher (0.99–2.92); this finding may be indicative of higher healthcare costs at a whole-population level for those in this category. HoNOS was originally designed for severely mentally ill populations,^6^ and it may not provide a good indicator of the presence of interpersonal and social problems among people diagnosed with personality disorder. Equally, it is possible that for people diagnosed with personality disorder, relationship and daily living problems are not associated with acute secondary health service use.

### Strengths and limitations

We examined routinely collected data from a large clinical population accessing secondary mental health services. Previous work has shown that the datalink between CRIS and HES is successful in 94% of cases.^13^ Nevertheless, our data were derived from health records not originally designed for research and may have contained coding errors typical of these sources. Furthermore, despite adjustment for confounders, residual confounding may have occurred. The distribution of data may have affected our regression models, with a high number of zeros, and cost may have been underestimated in our regression results. The cost data were a proxy measure for the type and intensity of secondary healthcare service use and were analysed using an epidemiological approach rather than a traditional health economic analysis. We only included acute secondary care service use without consideration of primary care costs, which may be considerable.^14^ A larger sample size may have produced estimates with narrower confidence intervals. This might be particularly relevant for the associations with severe agitation and aggression, where the lower limit of the confidence interval fell just below unity. Finally, we did not examine other markers of predictive validity, such as mortality or health-related quality of life; this may be a promising avenue for future research.

## Conclusions

Personality disorder severity is a key prognostic variable in routine clinical practice. The self-harm HoNOS domain, as routinely recorded in EHRs, appears to be a robust indicator of the severity of personality disorder, because it is prospectively linked to acute hospital health service use. Future research should explore the predictive validity of this indicator using other outcome measures.

## Data Availability

Data are available on reasonable request. The data accessed by CRIS remain behind a National Health Service firewall, and governance is provided by a patient-led oversight committee. Access to data requires approval by SLaM and an appropriate account set-up. Subject to these conditions, data access is encouraged, and those interested should contact the CRIS Administrator (cris.administrator@kcl.ac.uk).

## Supplementary material

For supplementary material accompanying this paper visit http://doi.org/10.1192/bjo.2023.509. click here to view supplementary material
