# Supplementary material for: Defining severity of personality disorder using electronic health records: short report
Source: BJPsych Open. 2023 Aug 1;9(5):e137. doi: 10.1192/bjo.2023.509 (PMC10486230; doi:10.1192/bjo.2023.509)
Supplement: Supplementary file 1 [file S2056472423005094sup001.docx]

**Defining severity of personality disorder using Electronic Health Records: Appendix 1**

**Methods**

*Further information on covariates*

Age was calculated at the time of first HoNOS rating. Years of follow up were calculated from first HoNOS rating to death or the end of the study period. Gender was categorized as female, male or other/not specified. Deprivation was calculated by linking participants’ address to Index of Multiple Deprivation (IMD). IMD scores were then split into tertiles. Ethnicity was recorded in participants’ electronic health records, and was then categorized into groups white, black and other. We acknowledge this is a crude grouping, but used this due to small numbers in sub groups. Co-morbid mood disorder was defined by ICD-10 code F30-, and psychosis by ICD-10 code F20-.

*Statistics*

Figure 1 shows the standardised residuals of the log transformed total healthcare cost.

**Figure 1: Standardised residuals from the regression of the log transformed total healthcare cost.**


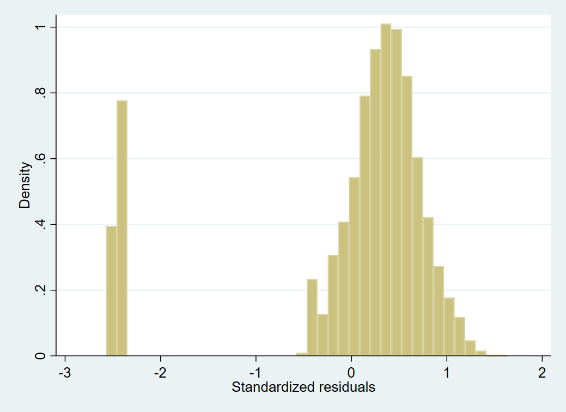


**Results**

Table 1 displays results from the linear regression examining the relationship between individual HoNOS items and the log transformed total healthcare cost, excluding individuals with zero cost. This model shows similar patterns to our main analysis. In this model there is no association between increasing severity of agitation and aggression and total healthcare cost.

Table 2 displays results from the linear regression examining the relationship between individual HoNOS items and the log transformed total healthcare cost, using a lower value for each inpatient bed day (£557.33; the average cost for a non-elective bed day). Table 3 displays results from the same analysis, using a higher value for each inpatient bed day (£1449.01; the average cost for an elective bed day). Both of these models showed similar results to our main analysis.

Results from the linear regression of the association between HoNOS items and total healthcare cost with bootstrapped standard errors are presented in Table 4. The model showed a similar trend to our main analysis for self-harm, and also found increasing daily living problems were associated with increased costs.

**Table 1. Linear regression of the association between HoNOS items and log transformed total healthcare cost, excluding individuals with zero cost**

|  |  |  | |  | | |  |
| --- | --- | --- | --- | --- | --- | --- | --- |
|  | **Adjusted coefficient** | | **95% confidence interval** | | | | **P** |
| **Agitation and aggression** |  | |  | |  |  |  |
| *Minor problem* | 1.03 | | 0.94 | | - | 1.13 | 0.526 |
| *Mild* | 1.01 | | 0.91 | | - | 1.13 | 0.813 |
| *Moderate* | 1.11 | | 0.97 | | -  -- | 1.28 | 0.131 |
| *Severe to very severe* | 1.01 | | 0.81 | | - | 1.26 | 0.920 |
| **Self-harm** |  | |  | |  |  |  |
| *Minor problem* | 1.05 | | 0.95 | | - | 1.17 | 0.316  0.316  0.316 |
| *Mild* | 1.25 | | 1.12 | | - | 1.40 | <0.001 |
| *Moderate* | 1.44 | | 1.25 | | - | 1.65 | <0.001 |
| *Severe to very severe* | 1.72 | | 1.45 | | - | 2.04 | <0.001 |
| **Relationship problems** |  | |  | |  |  |  |
| *Minor problem* | 0.93 | | 0.83 | | - | 1.05 | 0.241 |
| *Mild* | 0.89 | | 0.80 | | - | 0.99 | 0.039 |
| *Moderate* | 0.86 | | 0.76 | | - | 0.97 | 0.013 |
| *Severe to very severe* | 0.86 | | 0.71 | | - | 1.04 | 0.123 |
| **Daily living problems** |  | |  | |  |  |  |
| *Minor problem* | 1.06 | | 0.96 | | - | 1.16 | 0.253 |
| *Mild* | 1.29 | | 1.16 | | - | 1.43 | <0.001 |
| *Moderate* | 1.29 | | 1.07 | | - | 1.55 | 0.008 |
| *Severe to very severe* | 1.28 | | 0.87 | | - | 1.90 | 0.215 |

- **Reference category no problem.**
- Aggression n=5797, Self-harm n=5796, Relationships n=5776, Daily living n=5750.

**Table 2. Linear regression of the association between HoNOS items and log transformed total healthcare cost, with a cost of £557.33 for each inpatient bed day**

|  |  |  | |  | | |  |
| --- | --- | --- | --- | --- | --- | --- | --- |
|  | **Adjusted coefficient** | | **95% confidence interval** | | | | **P** |
| **Agitation and aggression** |  | |  | |  |  |  |
| *Minor problem* | 1.16 | | 0.90 | | - | 1.50 | 0.243 |
| *Mild* | 1.07 | | 0.79 | | - | 1.44 | 0.659 |
| *Moderate* | 1.38 | | 0.93 | | -  -- | 2.05 | 0.106 |
| *Severe to very severe* | 1.69 | | 0.99 | | - | 2.89 | 0.053 |
| **Self-harm** |  | |  | |  |  |  |
| *Minor problem* | 1.40 | | 1.06 | | - | 1.85 | 0.017 |
| *Mild* | 1.82 | | 1.33 | | - | 2.49 | <0.001 |
| *Moderate* | 1.75 | | 1.21 | | - | 2.54 | 0.003 |
| *Severe to very severe* | 2.63 | | 1.60 | | - | 4.31 | <0.001 |
| **Relationship problems** |  | |  | |  |  |  |
| *Minor problem* | 0.99 | | 0.73 | | - | 1.35 | 0.971 |
| *Mild* | 0.94 | | 0.70 | | - | 1.25 | 0.664 |
| *Moderate* | 0.91 | | 0.66 | | - | 1.27 | 0.592 |
| *Severe to very severe* | 1.05 | | 0.64 | | - | 1.71 | 0.849 |
| **Daily living problems** |  | |  | |  |  |  |
| *Minor problem* | 1.21 | | 0.94 | | - | 1.56 | 0.143 |
| *Mild* | 1.31 | | 0.98 | | - | 1.76 | 0.070 |
| *Moderate* | 1.17 | | 0.74 | | - | 1.86 | 0.491 |
| *Severe to very severe* | 1.82 | | 0.75 | | - | 4.43 | 0.187 |

- **Reference category no problem.**
- Aggression n=6613, Self-harm n=6611, Relationships n=6590, Daily living n=6563.

**Table 3. Linear regression of the association between HoNOS items and log transformed total healthcare cost, with a cost of £1449.01 for each inpatient bed day**

|  |  |  | |  | | |  |
| --- | --- | --- | --- | --- | --- | --- | --- |
|  | **Adjusted coefficient** | | **95% confidence interval** | | | | **P** |
| **Agitation and aggression** |  | |  | |  |  |  |
| *Minor problem* | 1.16 | | 0.89 | | - | 1.51 | 0.270 |
| *Mild* | 1.06 | | 0.78 | | - | 1.45 | 0.699 |
| *Moderate* | 1.38 | | 0.92 | | -  -- | 2.08 | 0.118 |
| *Severe to very severe* | 1.70 | | 0.98 | | - | 2.96 | 0.060 |
| **Self-harm** |  | |  | |  |  |  |
| *Minor problem* | 1.41 | | 1.06 | | - | 1.88 | 0.019 |
| *Mild* | 1.88 | | 1.36 | | - | 2.59 | <0.001 |
| *Moderate* | 1.86 | | 1.26 | | - | 2.73 | 0.002 |
| *Severe to very severe* | 2.90 | | 1.74 | | - | 4.85 | <0.001 |
| **Relationship problems** |  | |  | |  |  |  |
| *Minor problem* | 0.98 | | 0.71 | | - | 1.34 | 0.881 |
| *Mild* | 0.92 | | 0.68 | | - | 1.24 | 0.569 |
| *Moderate* | 0.89 | | 0.63 | | - | 1.25 | 0.496 |
| *Severe to very severe* | 1.03 | | 0.62 | | - | 1.71 | 0.919 |
| **Daily living problems** |  | |  | |  |  |  |
| *Minor problem* | 1.23 | | 0.95 | | - | 1.60 | 0.117 |
| *Mild* | 1.36 | | 1.01 | | - | 1.85 | 0.046 |
| *Moderate* | 1.26 | | 0.78 | | - | 2.03 | 0.347 |
| *Severe to very severe* | 2.01 | | 0.79 | | - | 5.10 | 0.142 |

- **Reference category no problem.**
- Aggression n=6613, Self-harm n=6611, Relationships n=6590, Daily living n=6563.

**Table 4. Linear regression of the association between HoNOS items and total healthcare cost with bootstrapped standard errors**

|  |  |  |  |  |
| --- | --- | --- | --- | --- |
|  | **Adjusted coefficient** | | **95% confidence interval** | **P** |
| **Agitation and aggression** |  | |  |  |
| *Minor problem* | 692.90 | | -2500.12 – 3885.93 | 0.671 |
| *Mild* | -382.48 | | -2770.900 – 2005.93 | 0.754 |
| *Moderate* | -631.34 | | -3409.30 – 2146.62 | 0.656 |
| *Severe to very severe* | 1047.85 | | -3799.67 – 5895.37 | 0.672 |
| **Self-harm** |  | |  |  |
| *Minor problem* | -1147.78 | | -3632.69 – 1337.13 | 0.365 |
| *Mild* | 3465.76 | | 375.93 – 6555.60 | 0.028 |
| *Moderate* | 5464.89 | | 1892.68 – 9037.11 | 0.003 |
| *Severe to very severe* | 4514.11 | | 468.85 – 8559.37 | 0.029 |
| **Relationship problems** |  | |  |  |
| *Minor problem* | -2270.47 | | -5281.85 – 740.92 | 0.139 |
| *Mild* | -2558.92 | | -5823.56 – 705.72 | 0.124 |
| *Moderate* | -4594.43 | | -7454.13 - -1734.72 | 0.002 |
| *Severe to very severe* | -2449.69 | | -7205.59 – 2306.22 | 0.313 |
| **Daily living problems** |  | |  |  |
| *Minor problem* | 1889.24 | | -517.67 – 4296.16 | 0.124 |
| *Mild* | 5057.56 | | 1982.64 – 8132.47 | 0.001 |
| *Moderate* | 5672.70 | | 1492.07 – 9853.32 | 0.008 |
| *Severe to very severe* | 12596.38 | | -2272.97 – 27465.73 | 0.097 |

- **Reference category no problem.**
- Aggression n=6613, Self-harm n=6611, Relationships n=6590, Daily living n=6563.
